# Learning and Transfer in Problem Solving Progressions

**DOI:** 10.3390/jintelligence10040085

**Published:** 2022-10-12

**Authors:** Jonathan S. Daniels, David Moreau, Brooke N. Macnamara

**Affiliations:** 1 Department of Psychological Sciences, Case Western Reserve University, Cleveland, OH 44106, USA; 2 Department of Psychology, University of Auckland, Auckland 1010, New Zealand

**Keywords:** learning, near-transfer, problem-solving, spatial reasoning, Rubik’s Cube

## Abstract

Do individuals learn more effectively when given progressive or variable problem-solving experience, relative to consistent problem-solving experience? We investigated this question using a Rubik’s Cube paradigm. Participants were randomly assigned to a progression-order condition, where they practiced solving three progressively more difficult Rubik’s Cubes (i.e., 2 × 2 × 2 to 3 × 3 × 3 to 4 × 4 × 4), a variable-order condition, where they practiced solving three Rubik’s Cubes of varying difficulty (e.g., 3 × 3 × 3 to 2 × 2 × 2 to 4 × 4 × 4), or a consistent-order condition, where they consistently practiced on three 5 × 5 × 5 Rubik’s Cubes. All the participants then attempted a 5 × 5 × 5 Rubik’s Cube test. We tested whether variable training is as effective as progressive training for near transfer of spatial skills and whether progressive training is superior to consistent training. We found no significant differences in performance across conditions. Participants’ fluid reasoning predicted 5 × 5 × 5 Rubik’s Cube test performance regardless of training condition.

## 1. Introduction

People are often challenged when having to learn new skills in a limited amount of time. In many cases, the most efficient way to learn a skill is to break it down to its core components and gradually increase complexity. For instance, when learning mathematics, we typically need to start with the basics, such as counting and addition before we move to progressively more complex functions such as multiplication or division. Likewise, when learning to read, we start by mapping sounds to written symbols before deciphering high-level construction. 

However, not all learning occurs from early, formal instruction like math and reading. Without guided instruction, we typically apply various solutions to new situations often making numerous errors (Ebbinghaus 1913). Slowly, patterns that lead to more directed and efficient manners of problem-solving are discovered (Fitts and Posner 1967). In a world where individuals are exposed to diverse and often unconstrained learning environments, such as when learning to solve problems of a spatial nature, it is important to consider how various learning progressions impacts one’s problem-solving abilities and overall performance. 

Learning, defined as our ability to encode new or modify old information and apply it to future situations, has been studied for generations (Gross 2010). One important component in the study of learning and memory is the transfer of learning—the idea that the concepts learned from one situation can be applied to another (Woodworth and Thorndike 1901). Numerous studies have shown that transfer of learning through training can improve one’s performance on more complex tasks (e.g., Broadbent et al. 2015; Meneghetti et al. 2017; Nayigizente and Richard 2000; Schiff and Vakil 2015; Vakil and Heled 2016). 

The transfer of learning can be divided into two types: near and far transfer. Near transfer—the application of learned situations to new, yet similar situations (as opposed to far transfer, which is associated with new and relatively different situations; Schunk 2004)—has often been studied for its application to spatial problems (e.g., Brunyé et al. 2019; Minear et al. 2016; Schiff and Vakil 2015; Trumbo et al. 2016; Vakil and Heled 2016). 

Spatial problem-solving is considered a key component in a number of performance domains from mathematics (Hegarty and Kozhevnikov 1999) to sports (Moreau et al. 2012). One of the tasks used to examine near transfer in spatial problem solving is the Tower of Hanoi (e.g., Helfenstein 2005; Minsky et al. 1985; Nayigizente and Richard 2000; Schiff and Vakil 2015; Tarasuik et al. 2017; Vakil and Heled 2016), a validated spatial reasoning task (Ahonniska et al. 2000). Of this literature, only a small percentage of studies has investigated training progressions, with designs systematically increasing levels of difficulty. An exception is Vakil and Heled’s (2016) work investigating the schema theory of discrete motor skill learning (Schmidt et al. 2011). The schema theory of discrete motor learning posits that schemas form as rules and parameters that are compared to novel situations. Based on this theory, Vakil and Heled (2016) hypothesized that participants would naturally search for generalizable solutions to the Tower of Hanoi, rather than a rigid and specific sequence of actions. To test this, they manipulated the order of training using the Tower of Hanoi, where some participants received towers varying in difficulties while others received the same level of difficulty. In support of the theory of discrete motor learning, Vakil and Heled (2016) found that participants in the varied training condition yielded better learning transfer than participants in the constant training condition. 

However, in spatial problem-solving tasks, it has yet to be tested what *type* of varied training leads to better transfer. In particular, the question remains whether varied training needs to be progressive in nature, where participants gain experience with incrementally more difficult versions of the task, or whether experiencing random variation is sufficient. Outside of spatial problem-solving, a working-memory training study (von Bastian and Eschen 2016) suggested that random variation may be sufficient. von Bastian and Eschen (2016) tested whether near transfer differed for participants randomly assigned to an adaptive, progression-ordered training, a randomly-varied training, a self-selected task difficulty training, or an active control. They found that training effects were not modulated by condition. They concluded that training with varying levels of task difficulty, regardless of progression order, produces training gains. 

von Bastian and Eschen (2016) were not the first to suggest that random variation may be beneficial for learning and perhaps more so than progressive sequences. Work by Shea and Morgan (1979) showed evidence of this by introducing difficulty in training a motor task via randomized order. Though participants in the randomized condition showed slower acquisition during training, they showed greater transfer and generalizability on later testing compared to their ordered-block counterparts. According to schema theory (Schmidt 1975; Wulf and Schmidt 1988), variability in practice is beneficial because it enhances the effectiveness of rules (schemata). Relatedly, Schmidt and Bjork (1992) argue that across verbal and motor tasks, variable practice encourages additional information processing leading to better transfer of learning. In this paper, we address the role of variable practice further from the domain of spatial problem-solving. 

### Present Study

The goal of the present study was to examine ordering effects in problem-solving progressions on a three-dimensional puzzle—the Rubik’s Cube. We chose to use three-dimensional puzzles rather than a computerized task to best examine Schmidt et al.’s (2011) theory of motor skill learning, and to increase the ecological validity of studying three-dimensional problems (Schiff and Vakil 2015; Vakil and Heled 2016).

We specifically chose the Rubik’s Cube for its difficulty. Whereas the Tower of Hanoi is fairly simple and training gains are easily found, only six percent of the global population has solved a Rubik’s Cube (Vega 2018). We thus sought to test different types of varied training in a task where participants would not be performing at ceiling with limited training. If we find converging evidence, this offers strong support for previous findings that used simpler tasks. In contrast, if we find countering evidence or no effects, this suggests that problem difficulty might be an important factor to consider when testing hypotheses about training and learning transfer in spatial problem solving.

Further, while previous research in this area has been based on motor skill learning, we were also interested in the pattern recognition and reasoning presumably active while attempting to solve Rubik’s Cubes. We assume that pattern recognition and reasoning are highly involved in Rubik’s Cube solving because Rubik’s Cubes have large problem spaces, which are defined as the number of possible states in the problem and the number of options at each stage of the solution (Newell and Simon 1972). As the problem space increases, so does task difficulty, and the presumed recruitment of cognitive resources (Frank and Macnamara 2021; Macnamara and Frank 2018). 

Finally, examining transfer effects with a novel paradigm, rather than with a common one from previous near-transfer literature, has the potential to give insight as to whether any effects from varied training is paradigm-specific or widespread across spatial reasoning problem-solving tasks. That is, the Rubik’s Cube not only differs from the Tower of Hanoi in the size of its problem space, but on the type of actions needed at each stage. The Tower of Hanoi involves moving independent objects (though with relational rules), whereas the Rubik’s Cube involves changing the task object, where each move shifts other components non-independently. 

We tested several hypotheses. One hypothesis is that participants in the progression-order group would perform significantly better on the final spatial problem-solving test than those in the variable-order group. This would support findings from complex learning studies where progression-order training is the norm (e.g., van Merriënboer and Kirschner 2017). Our competing hypothesis was that the variable-order group would have a similar level of performance on the final spatial problem-solving test as the progression-order group. This would support the schema theory of discrete motor skill learning and von Bastian and Eschen’s (2016) finding that solely varying the levels of task difficulty without adherence to a progression is as effective as progression-order training. 

Second, we hypothesized that both the progressive-order and variable-order conditions would outperform the consistent-order condition. We did not think it likely that participants in the consistent-order condition would outperform the other groups, as beginning training at a difficult level tends to slow learning (e.g., Ahissar et al. 2009). 

Finally, we postulated that participants’ fluid reasoning—one’s capacity to think logically and solve problems in novel situations, independent of acquired knowledge (Cattell 1987)—would be positively correlated with performance on the final test, across conditions. We also examined whether training condition moderated the relationship between fluid reasoning and test performance. 

## 2. Materials and Methods

Protocol and analyses were pre-registered, and materials and data can be found on the Open Science Framework, osf.io/uk6w2.

### 2.1. Participants 

An a priori power analysis indicated that a minimum of 128 participants was needed to test our hypotheses, assuming a medium effect size (*d* = 0.5) with .80 power and alpha set at .05. Our stopping rule was to collect data until the end of the week in which the 128th participant was recruited. A total of 141 members of the Case Western Reserve University community were recruited to participate in the study. Ninety-four participants were recruited through various advertisements throughout campus and compensated fifteen dollars at the completion of the study session. Forty-seven participants were recruited through the Case Western Reserve University’s Psychology subject pool for partial course credit in the Introduction to Psychology course. Eight participants who were recruited had successfully solved a Rubik’s Cube in the past three years and, following our pre-registered exclusion criteria, were excused from the study, and given partial course credit or five dollars. The final sample was 133 (84 female). The mean age was 24 years old (*SD* = 9.39 years). This study was approved by the Institutional Review Board at Case Western Reserve University.

### 2.2. Measures and Procedure 

Participants were brought into the lab and seated at one of six computer stations. In front of each participant were four Rubik’s Cubes, covered completely with the same-sized occluder such that participants did not know what was underneath and could not ascertain size differences. The occluders were bottomless boxes designed to cover Rubik’s cubes. The Rubik’s cubes remained covered until it was time to complete that puzzle. Thus, participants were unaware of an upcoming Rubik’s Cube complexity. 

Before revealing the Rubik’s cubes, participants were asked to complete the Raven’s Advances Progressive Matrices (RAPM; Raven 2003). The RAPM is a psychometrically-validated measure of fluid reasoning designed for adults with above-average reasoning. The RAPM is a multiple-choice test in which participants are tasked with completing matrix patterns by choosing the correct missing item. Odd-numbered items (18 total) of progressive difficulty were used and participants were given a ten-minute time limit to solve as many problems as they could. 

Following the RAPM, instructions appeared on the computer screen in front of the participant. Computer stimuli were created and presented using E-Prime 2 (Psychology Software Tools 2002) and displayed on a 23-inch OptiPlex 9030 computer screens. Stimuli were displayed at a resolution of 1920 × 1080 pixels at a refresh rate of 59 Hz.

The instructions on the computer screen read as follows: “In front of you, under each box is a puzzle. Your job is to solve each in order from left to right as best as you can. To solve each puzzle, you must successfully match all colors until there is only one distinct color on each side.” All participants had four minutes to independently work on each Rubik’s Cube before being prompted to move to the next, with thirty, twenty, and ten second warnings on the computer screen prior to the four-minute deadline. The four-minute timeline was based on pilot feedback. Greater amounts of time resulted in frustration and pilot participants indicating that they felt they would “undo” their progress by continuing beyond this amount of time.

**Figure 1:**
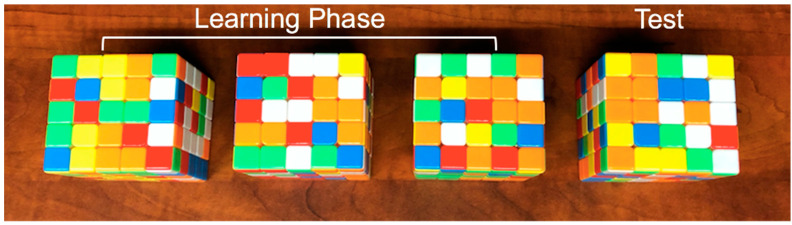
Consistent-order condition. Cube sizes are identical across the learning phase and at test (5 × 5 × 5).

**Figure 2:**
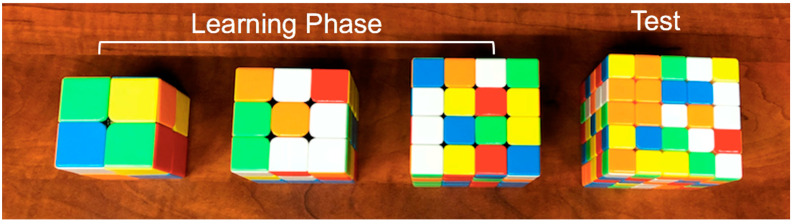
Progression-order condition. Cubes increase in size during the learning phase (2 × 2 × 2; 3 × 3 × 3; 4 × 4 × 4) before completing the test (5 × 5 × 5).

**Figure 3:**
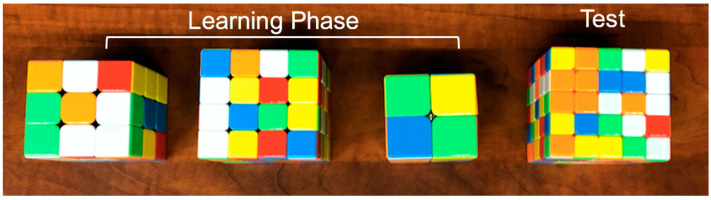
Variable-order condition example. Cubes vary in size during the learning phase (here, 3 × 3 × 3; 4 × 4 × 4, 2 × 2 × 2) before the test (5 × 5 × 5).

Participants were assigned to one of three conditions in counterbalanced order. In the progression-order condition, participants were tasked with solving increasingly difficult standardized Rubik’s Cubes under time restrictions (2 × 2 × 2 → 3 × 3 × 3 → 4 × 4 × 4). In the variable-order condition, participants were given the same time restrictions and were tasked with solving Rubik’s Cubes of varying difficulty in pseudo-random order (e.g., 3 × 3 × 3 → 2 × 2 × 2 → 4 × 4 × 4). (An order of 2 × 2 × 2 → 3 × 3 × 3 → 4 × 4 × 4 was excluded as it would be equivalent to the progression-order condition). In the consistent-order condition, participants had the same time restrictions to solve three 5 × 5 × 5 Rubik’s Cubes. Figure 1, Figure 2 and Figure 3 show the initial Rubik’s Cubes in each condition.

All participants were then given four minutes to solve a 5 × 5 × 5 Rubik’s Cubes as best as they could with thirty, twenty, and ten second warnings prior to the four-minute deadline. Scrambling of each Rubik’s Cube was based on algorithms generated from TNoodle-WCA-0.13.5, the official scrambling program for the World Cube Association (Fleischman et al. n.d.). Each participant received the same Rubik’s Cube configuration for each respective puzzle (i.e., all 2 × 2 × 2 puzzles were the same initial configuration, all 3 × 3 × 3 puzzles were the same configuration, etc.). Configurations can be found on Open Science Framework, osf.io/uk6w2. Finally, participants were asked to describe their thoughts and strategies during the tasks in an open-ended response using the computer keyboard, as well as indicate their gender and age on a computerized questionnaire.

### 2.3. Rubik’s Cube Scoring 

To measure performance on the Rubik’s Cubes, we recorded the maximum proportion of matching colors (green, blue, red, orange, white and yellow) on a single Rubik’s Cube side. For each color, we recorded the side with the highest number of that color. For example, a 3 × 3 × 3 Rubik’s Cube has nine squares on each of its six sides. If the participant had successfully matched a maximum of five red, two orange, four green, three blue, four white, and three yellow square pieces on any side, their completion would be recorded as 21/56 or 38%.

(1) $$\frac{\Sigma \;\left(\mathrm{maximum}\;\mathrm{number}\;\mathrm{of}\;\mathrm{like}\;\mathrm{colors}\;\mathrm{on}\;a\;\mathrm{side}\right)}{\mathrm{total}\;\mathrm{pieces}\;\mathrm{on}\;\mathrm{puzzle}}=\;\frac{5\left[\mathrm{red}\right]+2\left[\mathrm{orange}\right]+4\left[\mathrm{green}\right]+3\left[\mathrm{blue}\right]+4\left[\mathrm{white}\right]+3\left[\mathrm{yellow}\right]}{56\;\left[6*9\right]}=\frac{21}{56}$$

Percent completion does not always indicate better progress toward solving a Rubik’s Cube. That is, when solving a Rubik’s Cube, sometimes like colors need to be moved apart to make progress toward the solution. However, given that the Rubik’s Cubes could not be completely solved by novices in the timeframe given, participants were given the goal of moving as many like colors on the same side in the time allotted. Again, based on our piloting, the time chosen represented the time in which most pilot participants made the most progress toward this goal.

## 3. Results

Pre-registered analyses were as follows: (1) an independent samples *t*-test, comparing participants’ 5 × 5 × 5 Rubik’s Cube test completion between the progression-order and variable-order groups, (2) an equivalence test (Lakens 2017) to test the competing hypothesis that there was no difference on test performance between the progression-order and the consistent-order conditions, (3) an independent samples *t*-test comparing the progression-order and consistent-order groups, and (4) a Pearson’s correlation to test for a relationship between participants’ RAPM performance and their 5 × 5 × 5 Rubik’s Cube Completion test. We also pre-registered an exploratory moderator analysis and conducted an exploratory ANCOVA. Finally, we conducted additional Bayesian analyses to evaluate the evidence in favor of the null and alternate hypotheses. 

All priors used in the reported analyses are default prior scales (Morey et al. 2016). For the Bayesian ANCOVA, the prior scale on fixed effects was set to 0.5, the prior scale on random effects to 1, and the prior scale on the covariate to 0.354. The latter was also used in the Bayesian Linear Regression. All Bayesian *t*-tests used a Cauchy prior with a width of √2/2 (~0.707), meaning that half of parameter values are set to lie within the interquartile range [−0.707; 0.707]. In the next sections, we report these with robustness checks to quantify the influence of our choice of priors on the results.

### 3.1. Manipulation Check

To provide evidence of learning, we examined whether participants performed better on a given cube size if they received it later in their training sequence compared to participants who received the same sized cube earlier in their training. We found no significant differences in order of exposure for the 2 × 2 × 2 or the 3 × 3 × 3 cubes. However, participants who encountered the 4 × 4 × 4 cube at the end of the learning phase (whether in the progression-order or variable-order condition) outperformed participants who encountered the 4 × 4 × 4 cube first, *p* = .004, or second, *p* = .033.

#### 3.1.1. Progression-Order v. Variable-Order 

**Figure 4:**
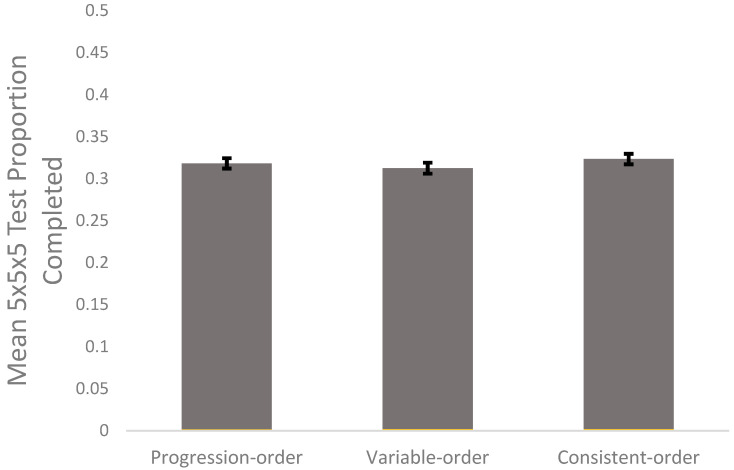
The 5 × 5 × 5 Rubik’s Cube mean completion in the progression-order (*n* = 44), consistent-order (*n* = 45), and variable-order (*n* = 44) groups. Error bars represent standard errors of the mean.

To test the hypothesis that the progression-order group would outperform the variable-order group on the 5 × 5 × 5 Rubik’s Cube test, an independent samples *t*-test was conducted using JASP (JASP Team 2018). This method was used instead of a one-way between-groups ANOVA because this hypothesis focused on a difference between these two conditions. There was no significant difference between progression-order test performance (*M* = 0.318, *SD* = 0.041) and variable-order test performance (*M* = 0.313, *SD* = 0.044), *p* = .558; *d* = 0.11 (see Figure 4).

**Figure 5:**
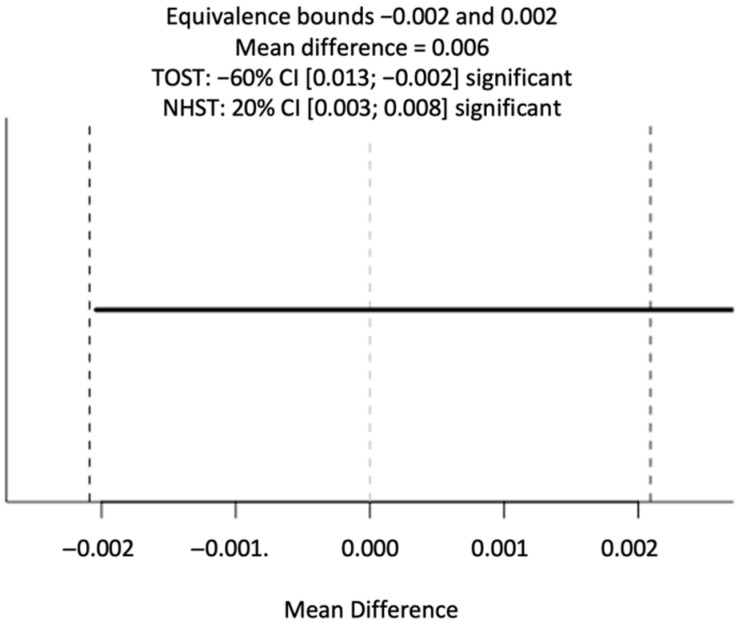
Results of the Independent Groups Student’s Equivalence test. The horizontal line indicates the confidence interval of the two one-sided *t*-test procedure, dotted vertical lines indicate equivalence bounds.

To test the competing hypothesis that there was no difference in test performance between the progression-order and the variable-order conditions, an equivalence test was conducted. Equivalence tests are conducted to test whether an observed effect is statistically smaller than the smallest effect size one would be concerned with, assuming there exists effects in a given population (Lakens et al. 2018). An Independent Groups Student’s Equivalence test was run in R-studio (Lakens 2017; RStudio Team 2016); forty-four participants in each condition, 0.80 power, and alpha set at .05. A mean difference of 0.006 supported statistical equivalence between the effects, suggesting that the conditions made a trivial difference in overall performance (see Figure 5).

**Figure 6:**
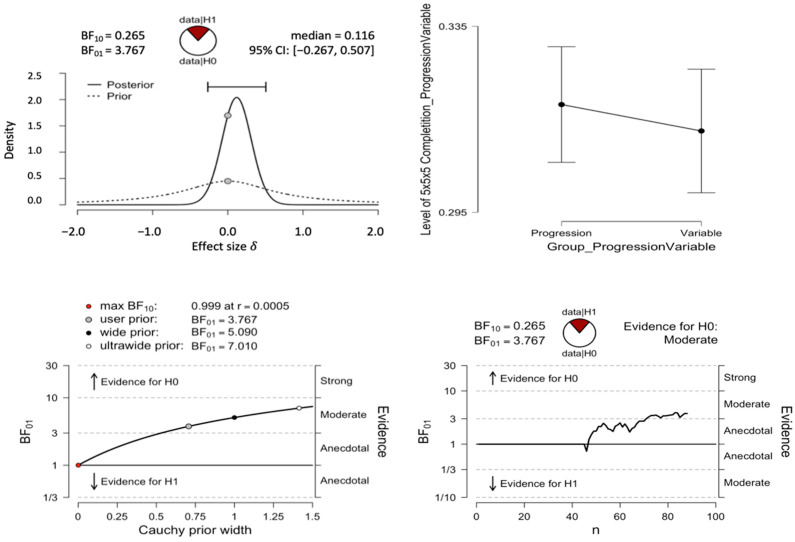
Results of Bayesian *t*-test for the progression-order and variable-order conditions. The top-left pane shows prior and posterior densities. The top-right pane shows the descriptive plot. The bottom-left pane shows the Bayes Factor Robustness check. The bottom-right pane shows the sequential analysis.

We also conducted a Bayesian independent samples *t*-test using JASP (JASP Team 2018). A Bayes factor of 3.77 with an error percentage of .033 was found in favor of the null hypothesis (see Figure 6).

#### 3.1.2. Progression-Order v. Consistent-Order

**Figure 7:**
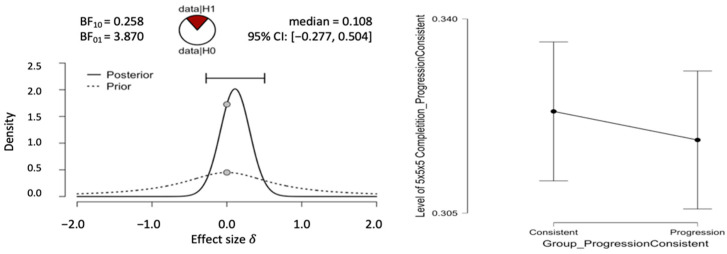

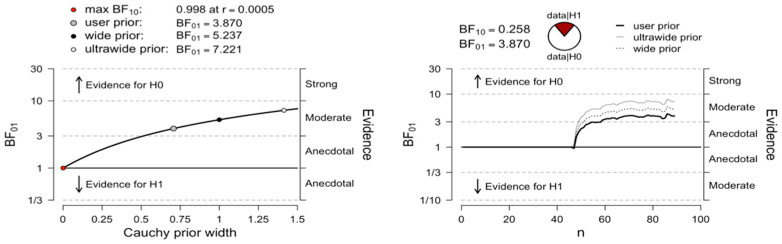
Results of Bayesian *t*-test for the progression-order and consistent-order groups. The top-left pane shows prior and posteriors densities. The top-right pane shows the descriptive plot. The bottom-left pane shows the Bayes Factor Robustness check. The bottom-right pane shows the sequential analysis.

To test whether the progression-order group outperformed the consistent-order group on the 5 × 5 × 5 Rubik’s Cube test, an independent samples *t*-test was conducted (JASP Team 2018). Again, these methods were used instead of a one-way between-groups ANOVA because this hypothesis focused on a difference between two conditions. There was no significant difference between progression-order test performance (*M* = 0.318, *SD* = 0.041) and consistent-order test performance (*M* = 0.323, *SD* = 0.042), *p* = .530, *d* = −0.13, (see Figure 5). A Bayes factor of 3.87 with an error percentage of .033 was found in favor of the null hypothesis (see Figure 7).

#### 3.1.3. Fluid Reasoning as a Predictor of Test Performance

**Figure 8:**
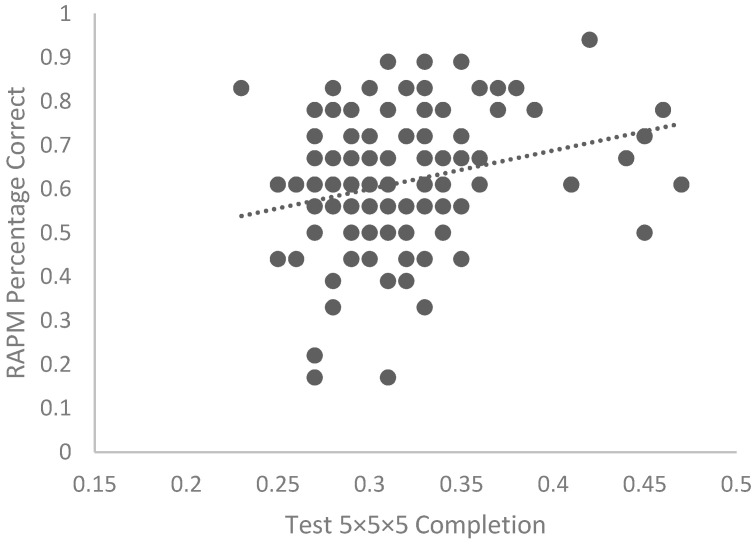
The 5 × 5 × 5 Rubik’s Cube Completion test and RAPM accuracy percentage across the full sample.

We tested the strength of the correlation between participants’ fluid reasoning as measured by the Raven’s Advance Progressive Matrices and their 5 × 5 × 5 Rubik’s Cube performance across the full sample. There was a weak, positive correlation, *r* = .24, *p* =.005, (see Figure 8) and a Bayes factor of 5.27 in favor of the alternative hypothesis—that fluid reasoning predicts Rubik’s Cube performance. 

### 3.2. Exploratory Analyses

In addition to the pre-registered analyses and the Bayesian analyses, we conducted two exploratory analyses. It could have been the case that variability in fluid reasoning was suppressing the effect of training order condition. To test whether 5 × 5 × 5 Rubik’s Cube completion depended on condition after controlling for fluid reasoning, we conducted with an ANCOVA where fluid reasoning served as the covariate. Condition did not emerge as a significant predictor after controlling for fluid reasoning *F*(2,129) = 0.62, *p* = .540. A Bayesian ANCOVA verified this result, producing a Bayes factor of 7.409 in favor of the null hypothesis.

We also tested whether fluid reasoning moderated the effectiveness of condition. Condition was dummy-coded with the consistent condition serving as the reference group, RAPM scores were centered, and dummy code × centered RAPM were created. Centered RAPM scores and dummy-coded conditions were entered into the model first and interactions were entered in the second step. Ravens and condition together explained 7% of the variance, *R*^2^ = .067, *p* = .029. *R*^2^ did not significantly change with the addition of the interactions (*R*^2^ change = .004, *p* = .776), indicating no significant moderation. A Bayesian Linear Regression verified this result, producing a Bayes factor of 1.597, *R*^2^ = .067, in favor of the null hypothesis.

## 4. Discussion

We sought to test the effects of the order of difficulty during training in a test of spatial problem solving and to test the influence of fluid intelligence on spatial problem solving. We first examined whether progression-order training yielded superior results to a variable-order condition on the final Rubik’s Cube performance, which would be in line with the methods of many progression-order training paradigms (e.g., van Merriënboer and Kirschner 2017) or whether there was no difference between progression-order training and variable-order training. Our results demonstrated no difference between the progression- and variable-order conditions. However, we also did not find that participants in these conditions outperformed participants in the consistent-difficulty condition, limiting the support our results offer for suggesting any variability is important (von Bastian and Eschen 2016) or for the schema theory of discrete motor skill learning, which also suggests participants should yield better transfer from varied versus consistent training.

Our results support our hypothesis that participants’ fluid reasoning would be positively correlated with performance on the spatial reasoning test, across conditions. The positive correlation found between fluid reasoning scores and 5 × 5 × 5 Rubik’s Cube completion suggests that cognitive abilities are important considerations for predicting overall performance on spatial problem-solving tasks, regardless of the training progression. 

Indeed, our results are in line with other studies that have found that spatial reasoning predicts performance but does not interact with the type of training. For example, Keehner et al. (2006, 2008), found that spatial reasoning ability predicted performance on a spatial rotation task regardless of the type of familiarization participants had with the stimuli (e.g., only seeing rotations of models versus interacting with the models, experienced versus novice laparoscopic surgeons). 

Considering the results of Vakil and Heled (2016), in which varied training on the Tower of Hanoi led to better schematic representation of the problem, we believe the lack of a superior method of learning may arise from the difficulty of the Rubik’s Cube. The Rubik’s Cube is substantially more complex than the Tower of Hanoi, and takes a large span of time to become familiar with; its complexities even evaded its inventor, Erno Rubik, who took nearly a month to solve it (Great Big Story 2017). 

In contrast to the Rubik’s Cube, the Tower of Hanoi is more easily solved because progress is easier to recognize (i.e., number of ascending size disks on the furthest right spoke). The Tower of Hanoi can be solved in 2*n* − 1 moves, where *n* equals the number of disks (Petković 2009). People can usually solve the Tower of Hanoi the first time they see it within a few minutes, and many can optimally solve it. In contrast, a person is unlikely to solve a scrambled Rubik’s Cube the first time they try, and optimally solving the Rubik’s cube requires the Hamiltonian path problem, a mathematical problem with a million-dollar prize for anyone able to solve it (Demaine et al. 2018; Ramachandran n.d.). While our lack of clear differences by condition might be due to needing longer training sessions on the Rubik’s Cube due to its difficulty, it may also be the case that varied training may be paradigm specific rather than widespread across spatial reasoning problems.

Despite limited findings, this paradigm has the potential to offer insight into the role of cognitive abilities on complex spatial problem-solving following training. Many studies investigating cognitive predictors of spatial task performance either use relatively simple laboratory tasks (e.g., the Tower of Hanoi) or use more complex real-world tasks where domain-specific knowledge and other confounds may be difficult to control (Keehner et al. 2006; Keehner et al. 2004; Minsky et al. 1985; Nayigizente and Richard 2000; Schiff and Vakil 2015; Tarasuik et al. 2017; Vakil and Heled 2016). These nuisance factors might be why findings in the literature are mixed. For example, Keehner et al. (2006) trained participants on using an angled laparoscope and found that spatial reasoning ability and general reasoning ability predicted performance initially, but only spatial reasoning ability continued to predict performance following twelve sessions of practice. However, another study by Keehner et al. (2004) found that spatial reasoning ability predicted performance following introductory laparoscopic training, but not following advanced laparoscopic training. In this study, participants differed in their surgical knowledge, with those taking the introductory training having limited surgical experience and those taking the advanced training having considerable surgical experience. Indeed, interactions between general cognitive ability and domain-specific knowledge have been found in other spatial tasks such as geological bedrock mapping (Hambrick et al. 2012), where visuospatial ability predicted performance among individuals with low geology knowledge, but not high geology knowledge.

## 5. Limitations and Future Directions

We designed our training paradigm to resemble those used in Tower of Hanoi training tasks and in von Bastian and Eschen’s (2016) working memory training tasks. That is, participants were given experience with variants of the task and then tested on a more difficult version of the task to examine near transfer. The trade-off with being consistent with these training paradigms is that we could not include a measure of baseline performance without interfering with the order conditions. Likewise, we did not provide strategy training on the Rubik’s Cube, which might have produced significant effects, but would not have been comparable to either Tower of Hanoi and in von Bastian and Eschen’s (2016) working memory training tasks. Strategy training may be a fruitful direction for future research.

The brief, i.e., four-minute time limits per puzzle, were chosen based on pilot performance and feedback from pilot participants (i.e., frustration levels). However, longer training sessions may be needed to observe improvements. Analogous studies of the Tower of Hanoi typically have no time restrictions for each training trial (Schiff and Vakil 2015; Vakil and Heled 2016). When time restrictions were imposed they were typically short, e.g., five-minute limits for puzzles that required only five moves to solve (Tarasuik et al. 2017). To study problem-solving progression with the Rubik’s Cube while avoiding participant frustration might therefore require a long-term study conducted over multiple sessions to provide insight into learning progressions using this paradigm. This approach may yield evidence for the schema theory of discrete motor skill learning as additional time may allow for participants to explore various possible solutions.

Longer training studies using the Rubik’s Cube paradigm could elucidate the role of the acquisition of domain-specific knowledge, which is difficult to control when observing real-world trainees. Likewise, domain-specific knowledge could be manipulated in a longer training paradigm. Domain-specific knowledge manipulations would allow researchers to examine interactions with cognitive abilities and types of training progressions, as well as how fluid reasoning might predict domain-specific knowledge acquisition rates, which in turn predict final performance. Further, longer training studies using this paradigm could shed light on the indirect role of cognitive abilities on spatial problem solving via influence on the rate of domain-specific knowledge acquisition. Understanding the role of cognitive abilities and various training conditions on a spatial problem-solving task could lead to better-informed training paradigms in domains such as sports, medicine, science, or engineering.

## Data Availability

Protocol and analyses were pre-registered, and materials and data can be found on the Open Science Framework, osf.io/uk6w2.
